# Material Properties of Titanium Diboride

**DOI:** 10.6028/jres.105.057

**Published:** 2000-10-01

**Authors:** Ronald G. Munro

**Affiliations:** National Institute of Standards and Technology, Gaithersburg, MD 20899-8520

**Keywords:** evaluated data, material properties, mechanical properties, physical properties, thermal properties, titanium diboride

## Abstract

The physical, mechanical, and thermal properties of polycrystalline TiB_2_ are examined with an emphasis on the significant dependence of the properties on the density and grain size of the material specimens. Using trend analysis, property relations, and interpolation methods, a coherent set of trend values for the properties of polycrystalline TiB_2_ is determined for a mass fraction of TiB_2_ ⩾ 98 %, a density of (4.5±0.1) g/cm^3^, and a mean grain size of (9±1) µm.

## 1. Introduction

Titanium diboride (TiB_2_) is well known as a ceramic material with relatively high strength and durability as characterized by the relatively high values of its melting point, hardness, strength to density ratio, and wear resistance [1]. Current use of this material, however, appears to be limited to specialized applications in such areas as impact resistant armor, cutting tools, crucibles, and wear resistant coatings. An important evolving application is the use of TiB_2_ cathodes in the electrochemical reduction of alumina to aluminum metal. Other applications may develop rapidly if the electrical discharge machining of TiB_2_ can be perfected. Broader application of this material may be inhibited by economic factors, particularly the cost of densifying a material with a high melting point, and concerns about the variability of the material properties. The present paper addresses the latter issue by examining the physical, mechanical, and thermal properties of TiB_2_ as a function of density and grain size.

This work extends the approach to data evaluation begun in previous studies on alumina [2] and silicon carbide [3]. The latter studies had a significant advantage over the present one, namely that the processing procedures were sufficiently well refined that batch to batch variations in the properties could be relatively small. For titanium diboride, the processing procedures do not seem to be as highly refined, and consequently, one must anticipate greater batch to batch variability. Therefore, it is all the more important to have a coherent view of the properties of TiB_2_ and their dependence on microstructure. The present work constructs such a view in the context of trends of property values.

The bane of all ceramic materials is that a particular measured property value for a particular specimen may depend on a particular feature of the particular microstructure of that particular specimen. In the absence of tightly controlled processing procedures, the best that one can do as a means of generically characterizing such a material is to establish trends of values that occur in correlation with changes in the microstructure and composition. Given such trends, it should then be possible to interpolate to a set of property values for a single constrained composition and microstructure such that the set is consistent both with respect to the trends and with respect to known mutual property relations. Fortunately, the trend of the value of a property across a range of microstructures depends on the statistical characterization of the microstructure. Therefore, the trend of a property value may have a discernable correlation with one or more statistics of the microstructure, such as mean grain size, mean pore size, or bulk density. This approach is applied here to the properties of TiB_2_.

## 2. Material Description

**Fig. 1:**
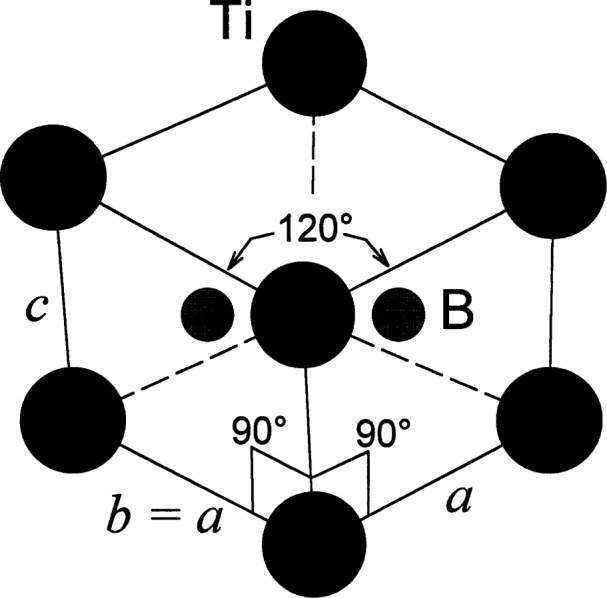
The hexagonal unit cell of single crystal TiB_2_, space group P6/mmm, *a* = *b*≠*c*, *α* = *β* = 90°, *γ* = 120°, 1 formula unit per cell, Ti at (0,0,0), B at (1/3,2/3,1/2) and (2/3,1/3,1/2).

**Fig. 2:**
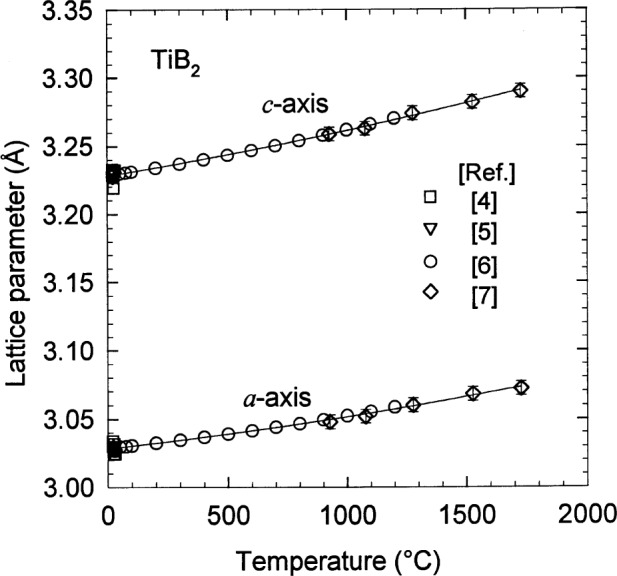
Lattice parameters *a* and *c* of single crystal TiB_2_ as a function of temperature.

Single crystal TiB_2_ exhibits hexagonal symmetry, Fig. 1 with space group P6/mmm. The lattice parameters [4–7], Fig. 2, have a slight quadratic dependence on the temperature which accounts for the linear temperature dependence of the coefficient of thermal expansion. The ratio *c/a* ranges from (1.066±0.001) at 25 °C to (1.070±0.001) at 1500 °C. Individually, the lattice parameters may be expressed as

$$a/\text{Å}=3.0236+1.73\times 10^{–5}\left(T/K\right)+3.76\times 10^{–9}{\left(T/K\right)}^{2}$$ (1)

$$c/\text{Å}=3.2204+2.73\times 10^{–5}\left(T/K\right)+3.95\times 10^{–9}{\left(T/K\right)}^{2}$$ (2)

where 293 K≤*T*≤2000 K, and the relative standard uncertainties [8] *u*_r_(*a*) = 0.03 % and *u*_r_(*c*) = 0.04 % are estimated from the variances of the least-squares fits. Using the molar mass *M* = 69.522 g/mol and the volume of the hexagonal unit cell *V* = (3/4)^1/2^ *a*^2^*c*, the density *ρ*_xtal_ of the single crystal can be calculated as

$$\rho_{\text{xtal}}=\frac{Mz}{N_{A}V},$$ (3)

where *z* = 1 is the number of formula units per unit cell, and *N*_A_ is the Avogadro constant. At 20 °C, *ρ*_xtal_ = (4.500±0.0032) g/cm^3^. The relative standard uncertainty *u*_r_(*ρ*_xtal_) is calculated as a propagation of uncertainty from the measured values of *a* and *c*; viz.

$$u_{r}{\left(\rho_{\text{xtal}}\right)}^{2}=u_{r}{\left(V\right)}^{2}=4u_{r}{\left(a\right)}^{2}+u_{r}{\left(c\right)}^{2}=5.2\times 10^{-7}.$$

Nearly fully dense polycrystalline TiB_2_ can be produced by a variety of processing methods, including sintering [9–13], hot pressing [14], hot isostatic pressing [10,11,15,16], microwave sintering [17], and dynamic compaction [18]. The relatively strong covalent bonding of the constituents, however, results in low selfdiffusion rates. Consequently, given also a high melting point of (3225±20) °C [19–21], pressureless sintering of TiB_2_ requires a relatively high sintering temperature, on the order of 2000 °C. Unfortunately, grain growth is also accelerated by the higher temperature, and the anisotropy of the hexagonal grain structure results in deleterious internal stresses and the onset of spontaneous microcracking during cooling. Grain growth can be limited and densification enhanced by the use of sintering aids such as Cr, CrB_2_, C, Ni, NiB, and Fe. The solubility of TiB_2_ in liquid Ni and Fe appears to be especially useful in this regard. In such cases, the mass fraction of the sintering aid in the specimen may range from 1 % to 10 %, while the sintering temperature may be reduced to the range of 1700 °C to 1800 °C for sintering times on the order of 1 h. Successful hot pressing with Ni additives can be achieved with a hot pressing temperature as low as 1425 °C with a sintering time of 2 h to 8 h [14]. When sintering aids are used in the composition, the theoretical maximum density, *ρ*_theo_, can be different from the density of the pure crystal, *ρ*_xtal_, because of the differing mass density of the sintering aid and the influence of the sintering aid on the lattice parameters.

## 3. Mechanical and Thermal Properties

The diversity of the processing conditions is a significant factor in the often widely varying property values reported in the literature for polycrystalline TiB_2_. In this section, the availiable mechanical and thermal properties are examined with the intent of providing a better understanding of how the properties depend on the composition, grain size, and density of the material.

### 3.1 Elastic Moduli

For isotropic polycrystalline materials, the elastic properties may be expressed in terms of two independent moduli, the elastic modulus *E* and the shear modulus *G*. Values of *E* [18, 22–26] determined at room temperature by ultrasonic velocity and resonance methods for various grain sizes and densities fall roughly into two groups that are distinguished by density, but which have little perceptible dependence on grain size. This observation is consistent with numerous models that consider elastic properties to vary principally as a function of porosity. Over a large range of porosity (as much as 50 %), the dependence is well described by an exponential model [27], *E* = *E*_s_ e^−^*^bϕ^*, although for lower degrees of densification the modulus decreases more rapidly [28]. In this expression, *E*_s_ and *b* are parameters, and *ϕ* is the volume fraction of porosity. For a wide variety of ceramics [29], *b ≈* 4.1±1.8. Hence, for porosity that is less than about 10 %, expanding the exponential to first order in *ϕ* yields *E ≈ E*_s_′ + *b*′ *ρ* using *ϕ* = 1−*ρ*/*ρ*_theo_ for total porosity and setting *E*_s_′ = *E*_s_ (1−*b*) and *b*′ = *E*_s_*b*/*ρ*_theo_. Consequently, it can be expected that the elastic modulus will be linear in the measured density.

**Fig. 3:**
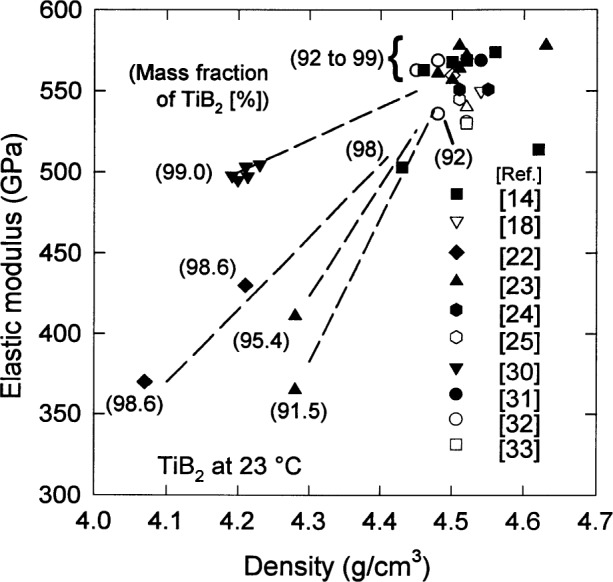
The elastic modulus of TiB_2_ at room temperature as a function of density.

**Fig. 4:**
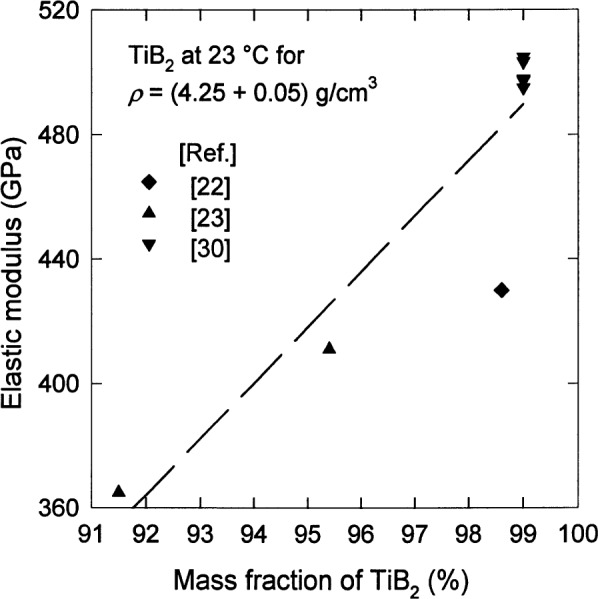
The significant increase in the elastic modulus of TiB_2_ with increasing mass fraction of TiB_2_ in the specimen at an approximately constant density.

**Fig. 5:**
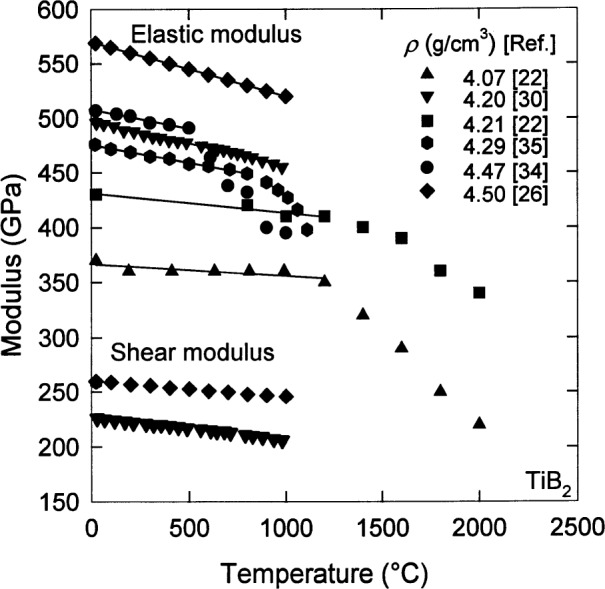
The temperature dependence of the elastic and shear moduli of TiB_2_ for various densities.

Neglecting any effect of the grain size in this case, Figs. 3 and 4 [14,18, 22–25, 30–33] show, respectively, that *E* has a significant dependence on both the density and the chemical composition. A higher mass fraction of TiB_2_ in the specimen yields a higher value of *E*. When the mass fraction of TiB_2_ in the specimen exceeds 90 %, the value of the elastic modulus appears to converge to 565 GPa at 23 °C as the density increases towards 4.5 g/cm^3^. At higher temperature *T* the value of *E* decreases as in Fig. 5 [22, 26, 30, 34–35], i.e., d*E*/d*T* < 0. While the value of *E* varies significantly with density and composition, the slope of *E* vs *T* is nearly constant with the mean value being d*E*/d*T* = −(0.032±0.015) GPa/K for temperatures less than 1000 °C. Consequently, for fully dense TiB_2_,

$$E=E_{0}+(dE/dT)(T-T_{0}),$$ (4)

where 296K⩽*T*⩽1273K, *E*_0_=565GPa, *T*_0_=296K, and *u*_r_(*E*)=5%.

The shear modulus, shown in Fig. 5 [26, 30–31] for two different densities, also varies linearly from room temperature to 1000 °C with the average slope being d*G*/d*T* = −(0.015±0.002) GPa/K. Hence, for fully dense TiB_2_,

$$G=G_{0}+(dG/dT)(T–T_{0}),$$ (5)

where 296K⩽*T*⩽1273K, *G*_0_=255GPa, *T*_0_=296K, and *u*_r_(*G*)=5%.

Poisson’s ratio (*v*) and the bulk modulus (*B*) can be calculated using the well known relations

$$v=\frac{E}{2G}-1$$ (6)

$$B=\frac{E\cdot G}{3(3G-E)},$$ (7)

which yield *v* = 0.11±0.08 and *B* = (240±57) GPa for fully dense TiB_2_ at room temperature.

### 3.2 Strength

**Fig. 6:**
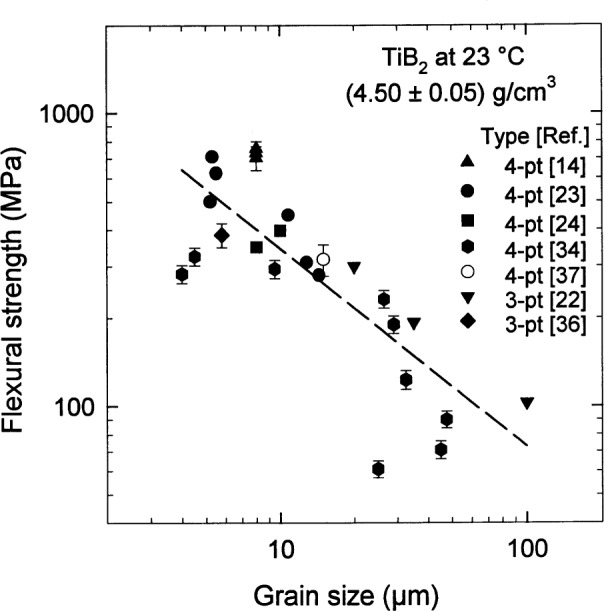
Flexural strength *σ*_f_ of TiB_2_ at room temperature as a function of grain size for a fixed density. The dashed line is a least-squares fit. Error bars are the reported standard deviations.

**Fig. 7:**
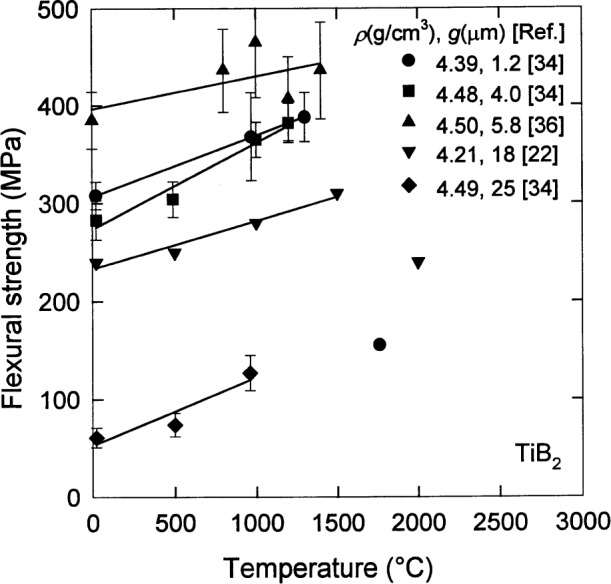
The temperature dependence of the flexural strength of TiB_2_ in three-point bending for various densities and grain sizes. The lines are least-squares fits for temperature less than 1500 °C. Error bars are the reported standard deviations.

Proceeding from the results of the previous subsection, let us restrict our attention for the moment to specimens with a density of (4.50±0.05) g/cm^3^ and consider the flexural strength *σ*_f_ of the material. A significant dependence on the grain size is readily seen in the results at room temperature shown in Fig. 6 [14, 22–24, 34, 36–37]. While this figure contains a mixture of data from three-point and four-point test methods using differing specimen sizes and crosshead speeds, the comparison clearly suggests that the strength *σ*_f_ decreases as the grain size increases. This result is at least consistent with reports that specimens prepared with grain size g > 15 µm exhibit spontaneous microcracking [14, 34, 38] in the microstructure which would tend to reduce the strength of the material. At elevated temperature, the slope of *σ*_f_ with respect to *T*, Fig. 7 [22, 34, 36], appears to be nearly constant for temperature less than 1500 °C and does not depend significantly on density, grain size, or test method. The average value of the slope is (∂*σ*_f_/∂*T*) = (0.06±0.02) MPa/K. Two effects have been suggested for the increase of strength with temperature. Strength may increase as a result of the relaxation of residual internal stresses produced in the specimens by the anisotropic thermal expansion of the microcrystalline constituent particles [34]. Crack healing due to oxidation and the formation of B_2_O_3_ may also contribute to this behavior for temperature up to about 1000 °C, but room temperature strengths of specimens oxidized at higher temperatures appear to be diminished by oxidation [36].

In general, the fracture strength of a brittle material is limited by microstructural inhomgeneities, commonly called flaws. Every batch of brittle specimens has a distribution of flaw sizes which results in a distribution of measured strength values. For most structural ceramics, the Weibull distribution with two parameters provides an adequate description of the strength distribution. In this distribution, the Weibull modulus parameter *m* provides an indication of the uniformity of the strength among the specimens. Higher values of *m* imply a narrower distribution of strengths. Reliable determinations of the Weibull modulus, however, require the fracture of a relatively large number of specimens, at least 30 specimens according to the ASTM (American Society for Testing and Materials) standard test method C 1161 [39]. For TiB_2_, results for this number of specimens have rarely been reported in the current literature. The three values that may be cited here have significant differences: *m* = 11 for a sintered material [24] with *ρ* = 4.55 g/cm^3^ and *g* = 8 μm; *m* = 29 for a hot pressed material [24] with *ρ* = 4.51 *g*/cm^3^ and *g* = 10 μm; and *m* = 8 for a hot pressed material [37] with *ρ* = 4.48 g/cm^3^ and *g* = 15 μm.

Like most structural ceramics, TiB_2_ is considerably stronger under compression than in flexure or tension. The quantity of available data is very limited, and no two results were obtained by the same method. With that caution, it appears, at room temperature, that the dependence of compressive strength *σ*_c_ on density is approximately linear, ranging [18, 22] from 1.1 GPa at 3.8 *g*/cm^3^ to 1.8 GPa at 4.5 g/cm^3^, when the grain size is (18±3) μm. There also appears to be a significant dependence on the grain size, but the data set is limited to only one additional value [24], 5.7 GPa for a density of 4.51 g/cm^3^ and a grain size of 10 μm.

### 3.3 Fracture Toughness

**Fig. 8:**
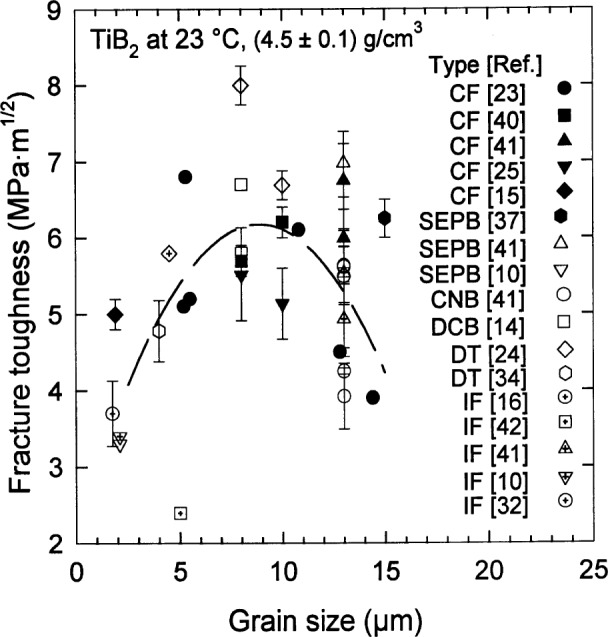
Fracture toughness *K*_Ic_ of TiB_2_ at room temperature as a function of grain size for a fixed density. Error bars are the reported standard deviations.

A clearer indication of the role of grain size in the optimization of the mechancial properties of TiB_2_ is provided by the fracture toughness as measured by the mode I critical stress intensity factor *K*_Ic_. For fully dense specimens at room temperature, having a mass fraction of TiB_2_ ⩾ 98 %, Fig. 8 [10, 14–16, 23–25, 32, 34,37, 40–42], *K*_Ic_ appears to have a maximum value for a mean grain size in the range 5 μm ⩽ *g* ⩽ 12 μm. The values in Fig. 8 may be influenced by three potentially significant factors: grain size, measurement method, and chemical impurity content. A statistical factor analysis of these data indicates that 75 % of the variability from the mean may be attributed to the variation of the mean grain size. The role of residual Ni impurities was considered explicitly in Ref. [14] and Ref. [23] where, neglecting the influence of grain size, it appeared that toughness increased with Ni content. However, taking into account the effect of grain size, the principal influence is seen to be microstructural rather than chemical. Combining this result with the observation in Fig. 6 that *g* < 10 μm is needed to optimize *σ*_f_, the optimum grain size for TiB_2_ should be in the range 5 μm ⩽ *g* ⩽ 10 μm. At the optimum, *K*_Ic_ = (6.2±0.5)MPa·m^1/2^.

### 3.4 Hardness

**Fig. 9:**
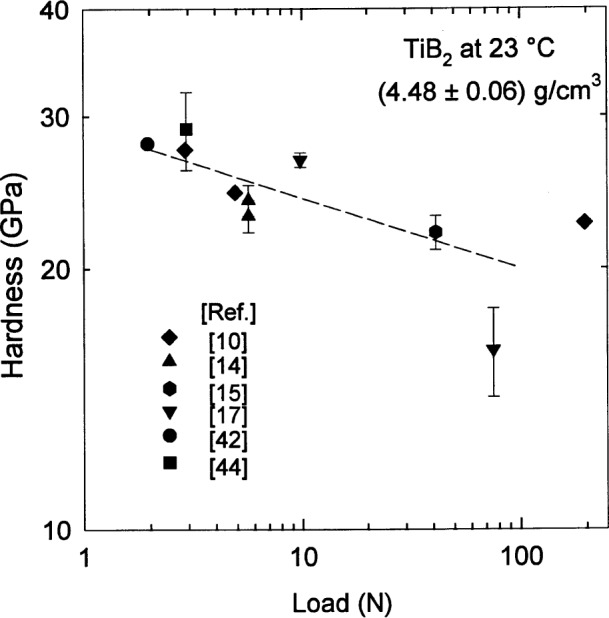
Hardness of TiB_2_ at room temperature as a function of indentation load for a fixed density.

Given the manner in which strength and toughness depend on density and grain size, it might be expected that the plastic deformation of the material under indentation would also exhibit a dependence on density and grain size. It is somewhat surprising, therefore, that a cursory examination of the data for the Vickers hardness [43]

$$H_{V}=1.8544\frac{P}{d^{2}}$$ (8)

of TiB_2_ has no immediately perceptible dependence on either density or grain size [10, 14, 15, 17, 42, 44]. *P* is the applied load and d is the length of the diagonal of the indentation impression. However, there is a significant scatter in the data that appears to be principally a consequence of measurement differences, particularly the use of different indentation loads, as shown in Fig. 9. The data in Fig. 9 are consistent with the indentation size effect [45] according to which the size of the diagonal length of the indentation impression is related to the applied load; this relation is often assumed to be in the form of the Meyer law [46, 47] which is expressed as

$$P=\zeta d^{\eta }$$ (9)

where *ζ* and *η* are parameters. Using a least-squares fit to the data in Fig. 9, it is easily found that *H* ∝ *P*^−0.08^, which corresponds to *η* = 1.85. Consequently, to assess density and grain size effects on hardness, we must simultaneously resolve the load dependence of the observed values.

To evaluate the simultaneous effects of density, grain size, and load, let us consider an empirical expression

$$H=h_{0}{\left(\frac{\rho }{\rho_{0}}\right)}^{h_{1}}{\left(\frac{g}{g_{0}}\right)}^{h_{2}}{\left(\frac{P}{P_{0}}\right)}^{h_{3}},$$ (10)

where the *h_i_* are adjustable parameters, and *ρ*_0_, *g*_0_,, and *P*_0_ are scale factors to make *h*_1_, *h*_2_, and *h*_3_ dimensionless. Applying Eq. (10) to the room temperature data, taking *ρ*_0_ = 4.5 g/cm^3^, *g*_0_ = 10 μm, and *P*_0_ = 10 N, yields *h*_0_ = 23 GPa, *h*_1_ = −4.1, *h*_2_ = −0.034, and *h*_3_ = −0.072, and the resulting fit has a relative uncertainty in the value of *H* of only 9 %.

**Fig. 10:**
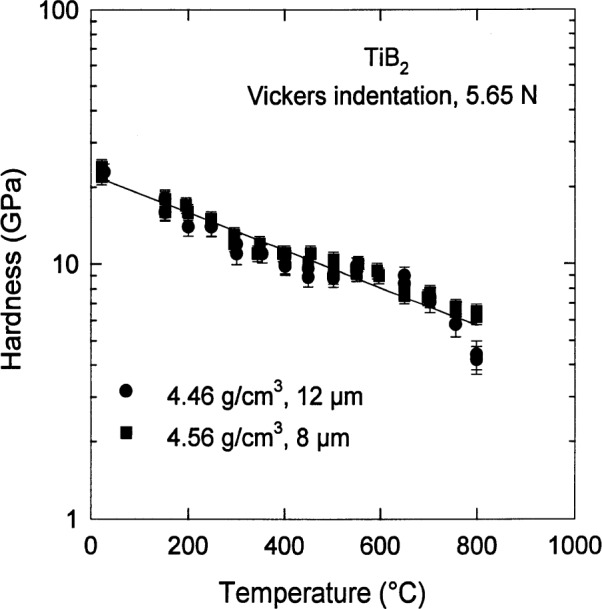
The temperature dependence of the hardness of TiB_2_.

With the precaution that the value of *H* depends on *ρ*, *g*, and *P*, the temperature dependence of the hardness [14] is shown in Fig. 10 for a load of 5.65 N and two conditions of density and grain size. As is often found for structural ceramics, *H*_V_ has an exponential dependence on temperature,

$$H_{V}=H_{0}\exp[-(T-T_{0})/\tau ],$$ (11)

where *H*_0_, *T*_0_, and *τ* are parameters. Taking *T*_0_ = 296 K, the value of *τ* can be found from Fig. 10 to be *τ* = 580 K. Using Eq. (10), the best estimate for *H*_0_ with *ρ*= 4.5 g/cm^3^, *g* = 10 μm, and *P* = 5 N is *H*_0_ = (24±2) GPa.

### 3.5 Creep

**Fig. 11:**
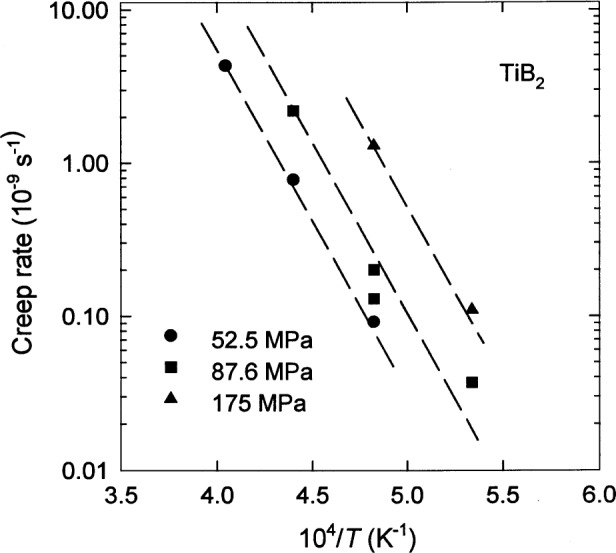
Flexural creep rate of TiB_2_ as a function of inverse temperature for various values of applied stress. The dashed lines show the fit of Eq. (12).

Deformation of a polycrystalline ceramic under sustained loading at high temperature produces creep, i.e., a strain that increases monotonically with time. A plot of strain vs time typically has three distinguishable regions denoted, respectively, as primary, secondary, and tertiary creep. While numerous mechanisms capable of producing creep have been identified [48], the principal mechanisms for creep in polycrystalline ceramics of high purity are thought to be solid state diffusional mechanisms. The secondary (also called steady-state) creep rate, d*ϵ*/d*t*, for diffusional [49] and dislocation [50] mechanisms is often expressed in the form of the Norton model [51]

$$\frac{d\epsilon }{dt}=A{\left(\sigma /\sigma_{0}\right)}^{n}\exp[-Q/RT],$$ (12)

where the amplitude factor *A*, the stress exponent *n*, and the apparent activation energy *Q* are adjustable parameters,*σ*_0_ is a fixed scale factor that may be taken to be 1 MPa, and *R* = 8.31451 J mol^−1^ K^−1^ is the molar gas constant. This model is valid for specimens with a constant grain size if log(d*ϵ*/d*t*) is linearly proportional to 1/*T* and if the plots for various fixed values of the applied stress *σ* are parallel. These conditions are satisfied approximately by the flexural creep data of TiB_2_ [22] as seen in Fig. 11. Applying Eq. (12) to these data, the parameters may be evaluated as *A* = 4.806×10^−4^ s^−1^, *n* = 2.3, and *Q* = 426 kJ/mol for *ρ* = 4.29 g/cm^3^ and *g* = 18 μm. With these parameters, the relative standard uncertainty of log(d*ϵ*/d*t*) is 20 % based on the statistical standard deviation of the fit.

### 3.6 Friction and Wear

Currently, one of the major uses of TiB_2_ is as a wear resistant material. For such applications, the friction and wear characteristics represent limiting benchmarks on the performance and durability of the material. In general, these characteristics are system properties, rather than material properties, and are functions of the temperature and loading conditions, the atmospheric and lubricating environments, the topological characteristics, and the relative sliding speed of the interacting surfaces [52]. However, in assessing the potential relative performance of materials in tribological applications, it is useful to know the friction and wear behavior of one specimen of the material sliding against another specimen of the same material in the absence of lubricating substances.

**Fig. 12:**
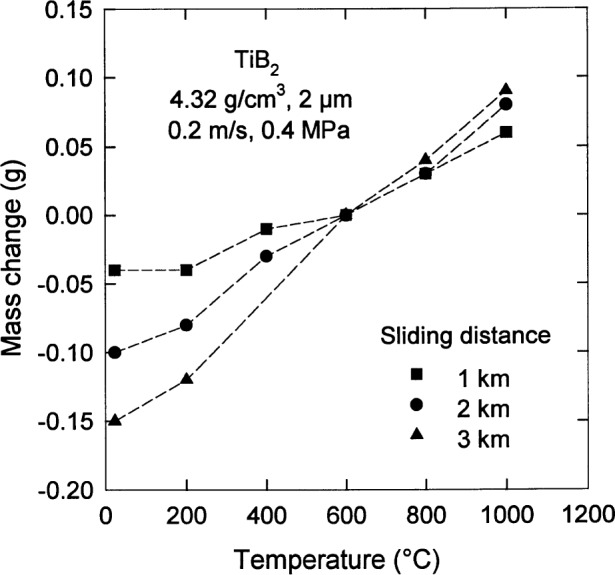
Wear results for TiB_2_ from a ring on block test as a function of temperature for various sliding distances with fixed values of the density, grain size, sliding speed, and applied load.

Even under such restricted conditions, the wear behavior of TiB_2_ is complicated by its interaction with oxygen in the atmosphere. Results from a ring on block test of the wear of TiB_2_ are shown in Fig. 12 [53] for a density of 4.32 g/cm^3^ and a grain size of 2 μm. For temperature less than 600 °C, the amount of material removed during the test increases with increasing sliding distance, but decreases with increasing temperature. For temperature greater than 600 °C, the specimens gain mass with the amount of mass gain increasing with increasing sliding distance. The decrease of mass loss and the occurence of mass gain appear to be the result of the formation of B_2_O_3_ in the wear track of the specimens.

**Fig. 13:**
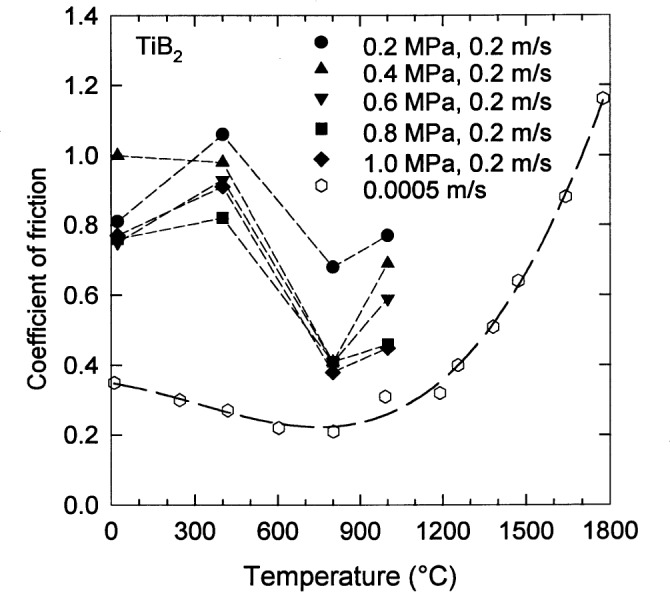
Coefficient of friction of TiB_2_ from ring on block tests for sliding speeds of 0.2 m/s (filled symbols, *ρ* = 4.32 g/cm^3^, *g* = 2 μm [53]) and 0.0005 m/s (open symbols, density and contact stress are unknown, *g* = 0.7 μm [54]).

**Fig. 14:**
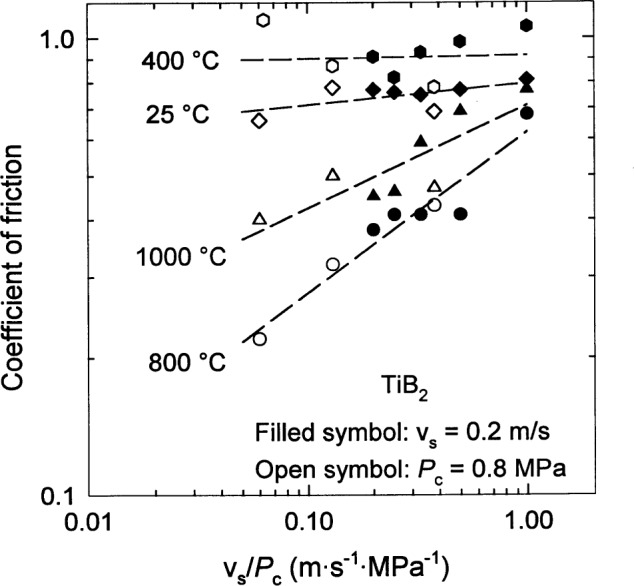
Dependence of the coefficient of friction of TiB_2_ on the ratio of sliding speed and contact stress at various temperatures.

The coefficient of friction [53, 54], Fig. 13, varies somewhat with temperature with an apparent minimum occurring for temperatures near 800 °C. The quantitative differences between the results of the two references are probably the result of different operating conditions in the two ring on block experiments. The coefficient of friction appears to have a power law dependence on the ratio of the sliding speed *v*_s_ and the contact stress *P*_c_, as seen in Fig. 14. At 800 °C, the friction coefficient has a value of about 0.2 when *v*_s_/*P*_c_ ≈ 0.06. In Ref. [54], the contact stresses were not reported, but the load was in the range of 0.25 N to 29.4 N (25 g to 3 kg). Hence, for the reported specimen dimensions, the apparent contact stress was in the range 1.4 kPa to 0.17 MPa, indicating that *v*_s_/*P*_c_ was in the range (0.36 to 0.003) m · s^−1^ · MPa^−1^, which is consistent with Fig. 14, though not conclusive. From Fig. 14, for *v*_s_/*P*_c_ = 0.2 m · s^−1^ · MPa^−1^, the coefficient of friction may be taken to be 0.8±0.1 for temperature less than or equal to 400 °C and 0.4±0.1 for temperature in the range 800 °C to 1000 °C.

A further characteristic of the wear process is provided by the dimensionless wear coefficient [55]

$$K_{W}=\frac{V_{W}H}{F_{n}D_{s}},$$ (13)

where *V*_w_ is the wear volume, *H* is hardness, *F*_n_ is the normal force acting between the surfaces, and *D*_s_ is the total sliding distance. For TiB_2_ at room temperature, *K*_w_ = (17±4)×10^−4^.

### 3.7 Specific Heat

**Fig. 15:**
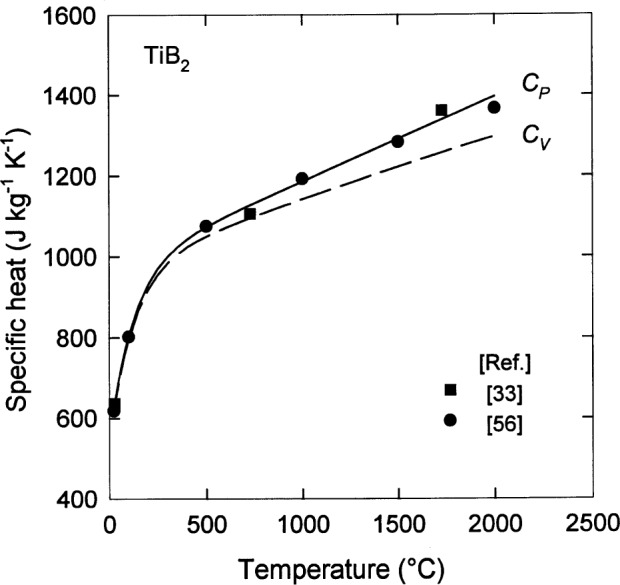
Specific heat *C_p_* of TiB_2_ as a function of temperature. The solid curve is the fit of Eq. (14). The dashed curve is *C_V_* calculated from Eq. (15).

There are several thermal properties that are important to most applications of ceramics at high temperature. The first of these is the specific heat, i.e., the amount of energy absorbed per unit mass to increase the temperature of the material by 1 K. For specimens with relatively high purity and density, this quantity is rather insensitive to variations in grain size or the presence of the small amounts of impurities. As shown in Fig. 15 [33, 56], the specific heat of TiB_2_ increases monotonically with increasing temperature. The rapid rise at low temperature and the linear variation at high temperature is readily fit by an interpolation formula of the form

$$C_{p}=c_{0}+c_{1}(T/K-273)+c_{2}\exp[-c_{3}(T/K-273)],$$ (14)

where *C*_p_ is the specific heat at constant pressure, and the parameters are *c*_0_=976J/(kgK), *c*_1_=0.21J/(kgK), *c*_2_=−426J/(kgK), and *c*_3_=0.008 for 293K⩽*T*⩽2273K. The relative standard uncertainty of the specific heat when these parameters are used with Eq. (6) is 1.5 % when the estimate of uncertainty is based on the standard deviation of the fit. Also shown in Fig. 15 is the specific heat at constant volume *C*_V_ which may be calculated from the thermodynamic relation

$$C_{P}-C_{V}=T\rho^{-1}B\;\alpha_{V}^{2},$$ (15)

where *α*_V_ is the mean volumetric coefficient of thermal expansion (CTE). For isotropic materials, *α*_V_ = 3 *α*_m_, where *α*_m_ is the mean linear CTE (Table 1, footnote h).

### 3.8 Thermal Transport

The transport of heat energy through the solid body of the material is described by two properties, thermal diffusivity *D* and thermal conductivity *κ*. Thermal diffusivity pertains to transient heat flow, while thermal conductivity pertains to steady state heat flow. The two properties are related such that

$$\kappa =\rho C_{P}D,$$ (16)

where *ρ* and *C_p_* are the density and specific heat, respectively.

**Fig. 16:**
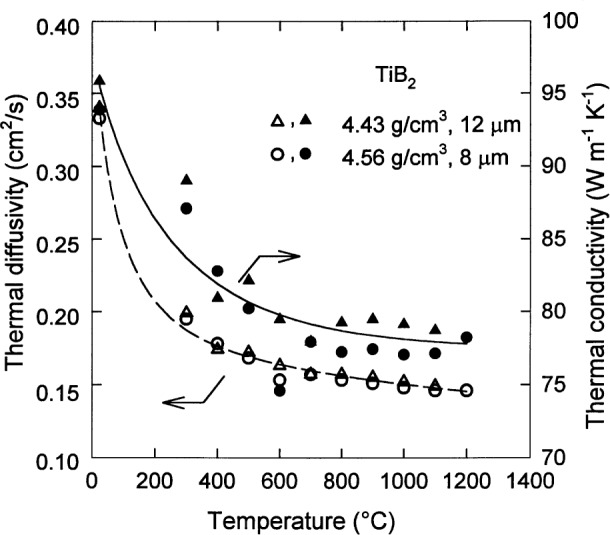
Thermal diffusivity D and thermal conductivity *κ* of TiB_2_ as a function of temperature. The dashed and solid curves are the fits of Eq. (17) and Eq. (18) respectively.

Data on the thermal transport properties of TiB_2_ are very scarce. Thermal diffusivity data [14] obtained using the laser flash technique are shown in Fig. 16 (open symbols) for two batches of TiB_2_. The higher density material with the smaller grain size also has a small nickel impurity (mass fraction of 0.43 %) which is not present in the other material. While it may be anticipated that these various factors may influence the diffusivity, there is insufficient data to discern any distinct effects at present. The diffusivity data can be converted to thermal conductivity data (filled symbols), using the results already given for density and specific heat. For each of these thermal transport properties, it is convenient to represent the values of the properties by an interpolation formula. For thermal diffusivity,

$$D=D_{0}\frac{D_{1}\exp[-D_{2}(T/K-273)]}{D_{3}+(T/K-273)},$$ (17)

where the *D*_i_ are adjustable parameters. The dashed curve in Fig. 16 is given by Eq. (17) when *D*_0_ = 0.145 cm^2^/s, *D*_1_ = 91.7 cm^2^/s, *D*_2_ = 0.00279, and *D*_3_ = 530. For thermal conductivity,

$$\kappa =\kappa_{0}+\frac{\kappa_{1}\exp[-\kappa_{2}(T/K-273)]}{\kappa_{3}+(T/K-273)},$$ (18)

where the *κ_i_* are adjustable parameters. The solid curve in Fig. 16 is given by Eq. (18) when *κ*_0_ = 77.3 W · m^−1^ · K^−1^, *κ*_1_ = 8270 W · m^−1^ · K^−1^ *κ*_2_ = 0.002, and *κ*_3_ = 410. With these parameters, the relative standard uncertainty in the value of either *D* or *κ* is 6 % in the temperature range 293 K≤*T*≤1473 K.

## 4. Conclusion

At the present time, there is no de facto production standard for TiB_2_, and consequently the variability of property values among different batches can be expected to be significant. However, trends in property values are related to the statistics of the microstructure, and that relation can be exploited to determine a consistent set of trend values for the properties of TiB_2_. Such trend values have been determined in the present work, Table 1, focusing on a particular density, *ρ* = (4.5±0.1) g/cm^3^, and mean grain size, *g* = (9±1) μm, as a function of temperature.

## Figures and Tables

**Table 1:** Mutually consistent trend values^a^ for properties of polycrystalline TiB_2_ deduced from the collection of observed particular values for specimens having mass fraction of TiB_2_ ⩾ 98 %, *ρ* = (4.5±0.1) g/cm^3^ and *g* = (9±1) μm, except as noted

Property	Temperature (°C)						
	20	500	1000	1200	1500	2000	*u*_r_^b^
Bulk modulus (GPa)	240	234	228				24
Compressive strength (GPa)	1.8						?
Creep rate^c^ (10^−9^ s^−1^)					0.005	3.1	20
Density^d^ (g/cm^3^)	4.500	4.449	4.389	4.363	4.322	4.248	0.07
Elastic modulus (GPa)	565	550	534				5
Flexural strength (MPa)	400	429	459	471	489		25
Fracture toughness (MPa · m^1/2^)	6.2						15
Friction coefficient^e^	0.9	0.9	0.6				15
Hardness (GPa)^f^	25	11	4.6				12
Lattice parameter^d^ *a*/Å	3.029	3.039	3.052	3.057	3.066	3.082	0.03
Lattice parameter^d^ *c*/Å	3.229	3.244	3.262	3.269	3.281	3.303	0.04
Poisson’s ratio	0.108	0.108	0.108				70
Shear modulus (GPa)	255	248	241				5
Sound velocity, longitudinal^g^ (km/s)	11.4	11.3	11.2				5
Sound velocity, shear^g^ (km/s)	7.53	7.47	7.40				3
Specific heat (J · kg^−1^ · K^−1^)	617	1073	1186	1228	1291	1396	1.5
Thermal conductivity (W · m^−1^ · K^−1^)	96	81	78.1	77.8			6
Thermal diffusivity (cm^2^/s)	0.30	0.17	0.149	0.147			6
Thermal expansion^d^,^h^ *α_a_*(10^−6^ K^−1^)	6.4	7.0	7.7	7.9	8.3	8.9	7
Thermal expansion^d^,^h^ *α_c_* (10^−6^ K^−1^)	9.2	9.8	10.4	10.6	11.0	11.6	5
Thermal expansion^h^ *α*_m_ (10^−6^ K^−1^)	7.4	7.9	8.6	8.8	9.2	9.8	6
Wear coefficient^e^ (10^−3^)	1.7						24
Weibull modulus^i^	11						?

a See text for relevant trend equations.

b Relative standard uncertainty (%); ? means insufficient information to determine u_r_.

c Flexure creep rate at 100 MPa, *ρ* = 4.29 g/cm^3^, *g* = 18 μm.

d Single crystal.

e *ρ* = 4.32 g/cm^3^, *g* = 2 μm, *v*_s_/*P*_c_ = 0.2 m · s^−1^ · MPa^−1^.

f Vickers hardness, load = 5 N.

g *v*_shear_ = (*G*/*ρ*)^1/2^; *v*_longitudinal_ = [(4/3) *G*/*ρ*+*B*/*ρ*]^1/2^.

h Coefficient of thermal expansion *α_x_* = (1/*x*_0_)(*x*−*x*_0_)/(*T*−*T*_0_), *x* = *a* or *c*, cumulative from the reference state at 20 °C(corresponding to *T*_0_ = 293 K); *α*_m_ = (2*α_a_*+*α_c_*)/3.

i Three reported values, 8, 11, and 29.
